# Oral health-related quality of life among long-term head and neck cancer survivors: a multinational study

**DOI:** 10.1007/s00520-025-09930-8

**Published:** 2025-09-20

**Authors:** Kristine Løken Westgaard, Cecilie Delphin Amdal, Katherine J. Taylor, Ragnhild Sørum Falk, Kristin Bjordal, Susanne Singer, Eva Hammerlid, Max Krüger, Carmen Stromberger, Orlando Guntinas-Lichius, Fréderic Duprez, Noa Stempler, Noam Yarom, Erofili Papadopoulou, Bente Brokstad Herlofson

**Affiliations:** 1 Department of Oral Surgery and Oral Medicine, Faculty of Dentistry, University of Oslo, Postbox 1109 Blindern, N-0317 Oslo, Norway; 2 Department of Otorhinolaryngology, Division for Head, Neck, and Reconstructive Surgery, Unit of Oral and Maxillofacial Surgery, Oslo University Hospital, Oslo, Norway; 3 Department of Oncology, Oslo University Hospital, Oslo, Norway; 4 Research Support Services, Oslo University Hospital Oslo, Oslo, Norway; 5 Institute of Medical Biostatistics, Epidemiology, and Informatics, University Medical Centre Mainz, Langenbeck, Germany; 6 Faculty of Medicine, University of Oslo, Oslo, Norway; 7 Department of Quality of Life in Oncology, Comprehensive Cancer Center Mecklenburg-Vorpommern (CCC-MV), University Medical Centre Rostock, Campus Rostock, Rostock, Germany; 8 Department of Otorhinolaryngology-Head and Neck Surgery, Institute of Clinical Sciences, Sahlgrenska Academy at University of Gothenburg, Sahlgrenska University Hospital, Gothenburg, Sweden; 9 Department of Oral and Maxillofacial Surgery - plastic Surgery, University Medical Centre Mainz, Mainz, Germany; 10 Department of Radiation Oncology, Charité-Universitätsmedizin Berlin, Corporate member of Freie Universität Berlin, Humboldt-Universität zu Berlin Germany, Berlin, Germany; 11 Berlin Institute of Health, Germany, Berlin Germany; 12 Department of Otorhinolaryngology, Jena University Hospital, Jena, Germany; 13 Department of Radiotherapy-Oncology, Ghent University Hospital, Ghent, Belgium; 14 Faculty of Medicine and Health Sciences – Department Human Structure and Repair, Ghent University, Ghent, Belgium; 15 Oral Medicine Unit, Sheba Medical Center, Tel Hashomer, Israel; 16 The Goldschleger School of Dental Medicine, Faculty of Medical and Health Sciences, Tel Aviv University, Tel Aviv, Israel; 17 Department of Oral Medicine and Pathology and Hospital Dentistry, Dental Oncology Unit, Dental School, National and Kapodistrian University of Athens, Athens, Greece

**Keywords:** Head and neck cancer, Oral health-related quality of life, Cross-sectional, Oral late effects, Survivor

## Abstract

**Purpose:**

Our aim was to investigate oral health-related quality of life (OHRQoL) and toxicities in long-term head and neck cancer (HNC) survivors diagnosed ≥five years earlier.

**Methods:**

HNC survivors treated between 2007 and 2013 participated in an international cross-sectional study. They completed the European Organization for Research and Treatment of Cancer (EORTC) core quality of life questionnaire (EORTC QLQ-C30) and the oral health module (EORTC QLQ-OH15) and attended a hospital examination. Clinicians scored toxicities using the Common Terminology Criteria for Adverse Events version 5.0.

OHRQoL was analyzed based on four types of treatment: surgery, radiotherapy, chemoradiotherapy without surgery and surgery with postoperative (chemo) radiotherapy. Survivors were divided into three groups according to the EORTC QLQ-OH15 oral health-QoL scale score; the lowest (defined as poor OHRQoL), middle and highest tertile.

**Results:**

Eleven sites in six countries enrolled 404 HNC survivors. The median time since diagnosis was 8.4 years, the mean age was 66 years and 67% were male. A total of 116 (29%) of the survivors reported poor OHRQoL. They were more often females and survivors with advanced disease. The survivors with poor OHRQoL also had more toxicity: dysphagia, trismus, osteonecrosis of the jaw, oral pain and dry mouth. There were no clinically significant differences in OHRQoL between the four treatment groups.

**Conclusion:**

Our study showed that the survivors who reported poor OHRQoL also had a high level of late toxicity. This highlight the need for improved follow-up of the oral health of HNC survivors many years after initial treatment, especially for women and those who were treated for advanced disease.

## Introduction

Head and neck cancer (HNC) represents a diverse group of malignancies and is the seventh most frequent cancer worldwide [1]. Approximately 932,000 new cases were diagnosed in 2020, and in the same year HNC was responsible for around 467,000 deaths. Overall, the five-year survival is 66% for the total group and has increased over the last decade, in particular for patients with loco-regional disease. Both the disease and treatment have significant impact on survivors’ lives causing decreased functioning and impaired health-related quality of life (HRQoL) [2, 3]. HRQoL is defined as a subjective and multidimensional concept of how disease and treatment affect a patient’s physical, emotional and social well-being (Quality of Life | EORTC – Quality of Life) [4, 5]. Even though the awareness and interest for oral health in cancer survivors are increasing, previous studies report lack of knowledge on oral late effects after cancer treatment [6, 7]. Oral health-related quality of life (OHRQoL) refers to the impact of oral symptoms and complaints on an individual’s general well-being and daily functioning [8]. More knowledge is needed about oral late effects in long-term HNC survivors, and on how these late effects may impact their OHRQoL. Patient-reported outcome measures (PROMs) are suitable for collecting information about patients’ experiences of symptoms, functioning and other aspects of HRQoL. Such information can be used to inform clinical practice. Use of PROMs to measure OHRQoL as a routine in clinical practice may have advantages in HNC patients, such as monitoring symptoms, assessing functional outcomes and evaluating treatment efficacy, leading to more informed clinical decisions and patient focused care [9].

Standard treatment of HNC includes surgery, radiotherapy (RT) and chemotherapy, often used in combinations [10, 11]. The side effects of HNC treatment may disturb several vital functions in the head and neck region such as chewing, swallowing and speech, and may cause nutritional problems, social isolation and reduced health-related quality of life (HRQoL) [12]. Surgery can lead to loss of important structures, scarring and deformities in the head and neck region, whereas chemotherapy may cause nausea, fatigue and mucositis [13]. RT is associated with mucositis, dysphagia, trismus, sticky saliva, dry mouth and risk of osteoradionecrosis [14, 15]. Several studies have assessed acute oral adverse effects and OHRQoL during treatment and first year after diagnosis [16–19] and other studies have shown that HNC survivors experience several oral late effects beyond the first year [20–22]. After five years post-treatment, HNC survivors are not routinely followed by specialists and little data on long-term OHRQoL effects is available [23]. The number of survivors included in existing studies is limited, despite that almost two-thirds of the survivors will reach the 5-year milestone [24–27].

The present study aimed to provide more knowledge on long-term OHRQoL in a multinational HNC population more than five years post-treatment. We aim to describe the survivors OHRQoL in relation to treatment, and the characteristics of survivors with poor OHRQoL, in order to better identify and manage patients at risk for severe oral late effects.

## Methods

### Study design, inclusion and exclusion criteria

This study was conducted as part of the European Organization for Research and Treatment of Cancer (EORTC) project “Late Toxicity and Long-term Quality of Life in Head and Neck Cancer Survivors” (EORTC 1629) [28]. The EORTC 1629 project is an international cross-sectional study carried out by members of the Quality of Life Group and the HNC Group of the EORTC, with the aim to assess long-term quality of life and toxicities in survivors of HNC. Eligible survivors treated between 2007 and 2013 were invited to complete HRQoL-questionnaires and undergo a clinical examination with toxicity assessment and an oral health assessment. Eligibility criteria were age ≥18 years, confirmed HNC diagnosis at least five years in the past and ability to attend the clinical visit, and completed validated questionnaires in one of the available languages. Survivors were recruited from 11centers in six countries: Norway, Sweden, Germany, Belgium, Israel and Greece from October 2018 to October 2021. Survivors who fit the inclusion criteria at each site were identified by date of diagnosis, and asked to participate by mailed invitation letters, at a follow-up appointment in hospital, or by telephone. Of 1105 HNC survivors in the EORTC 1629 study, 407 completed the oral HRQoL assessment needed for inclusion in the current study. Three survivors were excluded due to missing treatment information (*n*=1) or missing questionnaires (*n*=2). Thus, 404 survivors were included in the analysis sample. The ethical approval did not permit the collection of data on individuals who declined participation.

### Collection of demographic and clinical data

At each center, clinicians filled in a Case Report Form either electronically or on paper, to record information on survivors’ sex, age, country, living situation, length of education, smoking status, diagnosis, time since diagnosis and HNC treatment received. Clinical data including Karnofsky performance status and comorbidity according to the Charlson comorbidity index were obtained from the clinical examinations and from medical records [29]. Clinicians reported UICC stage using both version 7 and 8, but all Tumor Node Metastasis (TNM) values were converted to the 7^th^ edition of the UICC classification for simplification reasons [30]. Clinicians rated toxicity according to the Common Terminology Criteria for Adverse Events (CTCAE) version 5.0 [31]. Data from five oral toxicities (dysphagia, trismus, osteonecrosis of the jaw, oral pain and dry mouth) were used in this sub-study on oral health.

The survivors were categorized/divided into four treatment groups: Surgery only (including surgery of the primary tumour and additional neck dissection), RT only, chemo-radiotherapy (CRT) without surgery, and surgery with postoperative RT ± chemotherapy.

### Questionnaires

Participants completed the validated EORTC quality of life core questionnaire (EORTC QLQ-C30 V3) [32, 33] and the oral health module (EORTC QLQ-OH15) [8] in their own mother tongue before they attended a clinical examination at the hospital. The EORTC QLQ-C30 is a cancer specific 30-item HRQoL questionnaire that consists of five functional scales, nine symptom scales and one global quality of life scale. The EORTC QLQ-OH15 is a 15-item module focusing on OHRQoL in cancer patients. It comprises an eight-item scale named OH-QoL, in addition to seven single-items focusing on problems with dentures, sore mouth, sticky saliva, sensitivity to food and drink and satisfaction with information received.

Both questionnaires use a 4-point Likert scale with response categories from 1 = "not at all" to 4 = "very much". The responses are linearly transformed to a score between 0-100 for each scale. For functional scales in the EORTC QLQ-C30, higher scores indicate better functioning. In the EORTC QLQ-C30 and EORTC QLQ-OH15 symptom scales, higher scores indicate more problems. In the EORTC QLQ-OH15, higher OH-QoL scale scores indicate better oral health while higher scores on the information scale indicate more satisfaction with information received.

### Statistical analysis

The HNC survivors were divided into tertiles according to the EORTC QLQ-OH15 OH-QoL scale score; lowest (<75), middle (75-90) and highest (>90), where survivors in the lowest tertile were defined as having poor OHRQoL. The tertiles aimed to be equally sized, however, the groups were not perfectly equal due to ties (*n* = 116, 139 and 149). The characteristics of the HNC survivors were presented for the total population, by treatment groups, and by tertiles of the OH-QoL scale. Data were presented as frequencies and proportions for categorical data, and as mean, standard deviation (SD) and range for continuous data. Differences in characteristics between groups were tested by analysis of variance (ANOVA), Fisher’s exact test, Chi-square test, or Jonckheere–Terpstra test for trend, as appropriate.

The summary statistics (mean with 95% confidence interval (CI) and SD) for all scales in the EORTC QLQ-C30 and EORTC QLQ-OH15 were reported separately for each treatment group, together with the number of missing values. For the OH-QoL scale and single items, differences in the mean scores across treatment groups were explored using analysis of covariance (ANCOVA). A difference of 10 points between treatment groups were considered as minimal important difference (MID) and clinically significant [34]. To account for multiple testing, Tukey post-hoc test was performed. In multivariable models, age at survey, sex, UICC stage and tumour site (oropharynx/oral cavity/others) were included as co-variables.

Logistic regression analyses were performed to explore factors associated with poor OHRQoL compared to middle and high OH-QoL score combined. The multivariable analysis included age at survey, sex, tumour site (oropharynx/oral cavity/others), treatment group and time since diagnosis. UICC stage was omitted due to its correlation with treatment. Considering the multinational design of the study, we allowed for intragroup correlations by country. Results were presented as odds ratios (OR) with 95% CIs and p-values.

All analysis were carried out using Stata (StataCorp. 2023. Stata Statistical Software: Release 18. College Station, TX: StataCorp LLC).

## Results

### Survivor characteristics by treatment groups

Of the 404 HNC survivors, the mean age at survey was 66 years (range 23-91) and 67% were men (Table 1). The most frequent tumour subsites were oropharynx (42%) and oral cavity (27%). Most of the survivors had been treated with CRT without surgery (*n* = 155, 38%) or surgery with postoperative RT ± chemotherapy (*n* = 151, 37%). Survivors with oropharynx cancer mostly had received CRT (62%) or surgery combined with RT ± chemotherapy (22%), while most of those treated for oral cancer had received surgery in combination with RT ± chemotherapy (62%) or surgery only (26%). The median time since diagnosis was 8.4 years for all treatment groups.

**Table 1:** Characteristics of long-term head and neck survivors by treatment

	Total *n* = 404	Surgery *n* = 57	RT *n* = 41	CRT *n* = 155	Surgery + RT ± CT *n* = 151
	*n* (%)	*n* (%)	*n* (%)	*n* (%)	*n* (%)
Sex					
Male	271 (67)	34 (60)	27 (66)	117 (75)	93 (62)
Female	133 (33)	23 (40)	14 (34)	38 (25)	58 (38)
Age at survey (years)^a^					
Mean [SD] (range)	66 [10] (23-91)	68 [11] (33-91)	70 [8] (50-89)	65 [8] (45-86)	65 [11] (23-88)
Country^c^					
Norway	148 (37)	8 (14)	10 (24)	67 (43)	63 (42)
Sweden	101 (25)	19 (33)	12 (29)	48 (31)	22 (15)
Germany	75 (19)	20 (35)	1 (2)	11 (7)	43 (28)
Belgium	63 (16)	8 (14)	18 (44)	22 (14)	15 (10)
Israel	10 (2)	2 (4)	0 (0)	3 (2)	5 (3)
Greece	7 (2)	0 (0)	0 (0)	4 (3)	3 (2)
Living situation					
Together with others	299 (74)	36 (63)	29 (71)	114 (74)	120 (79)
Alone	99 (25)	20 (35)	12 (29)	38 (25)	29 (19)
Missing	6 (1)	1 (2)	0 (0)	3 (2)	2 (1)
Length of education					
≤10 years	147 (36)	17 (30)	23 (56)	48 (31)	59 (39)
>10 years	254 (63)	39 (68)	18 (44)	106 (68)	91 (3660)
Missing	3 (1)	1 (2)	0 (0)	1 (1)	1 (1)
Smoking status					
Never smoker	141 (35)	24 (42)	10 (24)	59 (38)	48 (32)
Former smoker	218 (54)	27 (47)	30 (73)	84 (54)	77 (51)
Current smoker	42 (10)	5 (9)	1 (2)	11 (7)	25 (17)
Missing	3 (1)	1 (2)	0 (0)	1 (1)	1 (1)
Tumour subsite^b^					
Oropharynx	170 (42)	10 (18)	17 (41)	106 (68)	37 (25)
Oral cavity	110 (27)	29 (51)	1 (3)	12 (8)	68 (45)
Larynx	40 (10)	5 (9)	16 (39)	7 (5)	12 (8)
Hypopharynx	9 (2)	1 (2)	4 (10)	4 (3)	0 (0)
Nasopharynx	16 (4)	0 (0)	1 (2)	14 (9)	1 (1)
Nasal cavity/sinuses	10 (2)	0 (0)	0 (0)	2 (1)	8 (5)
Parotid/other salivary glands	27 (7)	4 (7)	0 (0)	0 (0)	23 (15)
Unknown primary	22 (5)	8 (14)	2 (5)	10 (6)	2 (1)
Histology^c^					
Squamous cell carcinoma	353 (87)	50 (88)	39 (95)	150 (97)	114 (76)
Other	46 (11)	6 (11)	2 (4)	2 (1)	36 (24)
Missing	5 (1)	1 (2)	0 (0)	3 (2)	1 (1)
UICC stage^c^					
I	79 (20)	29 (51)	15 (37)	0 (0)	35 (23)
II	62 (15)	8 (14)	12 (29)	7 (5)	35 (23)
III	113 (28)	12 (21)	6 (15)	64 (41)	31 (21)
IV	139 (34)	7 (12)	7 (17)	80 (52)	45 (30)
Missing	11 (3)	1 (2)	1 (3)	4 (3)	5 (3)
Karnofsky Performance Status					
100	193 (48)	33 (58)	12 (29)	76 (49)	72 (48)
80 and 90	176 (44)	21 (37)	25 (61)	66 (43)	64 (42)
≤70	31 (8)	2 (4)	4 (10)	12 (8)	13 (9)
Missing	4 (1)	1 (2)	0 (0)	1 (1)	2 (1)
Charlson comorbidity index					
0	246 (61)	36 (63)	22 (54)	99 (64)	89 (59)
1	85 (21)	13 (23)	3 (7)	31 (20)	38 (25)
2	41 (10)	6 (11)	7 (17)	16 (10)	12 (8)
≥3	32 (8)	2 (4)	9 (22)	9 (6)	12 (8)
Current evidence of disease					
Yes	4 (1)	1 (2)	1 (2)	0 (0)	2 (1)
No	397 (98)	54 (95)	40 (98)	155 (100)	148 (98)
Missing	3 (1)	2 (4)	0 (0)	0 (0)	1 (1)
Time since diagnosis					
5 to < 8 years	142 (35)	17 (30)	21 (51)	61 (39)	43 (28)
8 to < 10 years	147 (36)	22 (39)	13 (32)	55 (36)	57(38)
≥ 10 years	115 (28)	18 (32)	7 (17)	39 (25)	51 (34)

^a^ Analysis of variance (ANOVA) *p* < 0.005

^b^ Fisher’s exact *p* < 0.005

^c^ Chi-square test for independence *p* < 0.005

*RT* Radiotherapy

*CT* Chemotherapy

*CRT* Chemo-radiotherapy

*SD* Standard deviation

*UICC* Union for International Cancer Control

### OHRQoL results

Survivors in the surgery only group reported less sticky saliva (mean 21.6, 95% CI 14.6-28.6) than survivors in the other treatment groups in unadjusted analyses (Table 2). The difference between groups remained clinically significant after adjustment. However, there were no statistically significant differences between groups in multivariable analyses after adjustment for multiple testing. Survivors who had received surgery only reported less sensitiveness to food and drink (mean 14.9, 95% CI 6.2-23.6) and survivors treated with RT only reported more soreness in the mouth (mean 30.0, 95% CI 21.4-38.6), compared to the other treatment groups. Survivors who had received RT only, reported most problems with ill-fitting dentures (mean 10.3 95% CI −6.5-27.0).

**Table 2:** OHRQoL measured with the EORTC QLQ-OH15 according to the type of treatment received – all scales and items, observed values

	OHRQoL	Totals	Surgery	RT	CRT	Surgery + RT ± CT
		Mean (95%CI)	Mean (95%CI)	Mean (95%CI)	Mean (95%CI)	Mean (95%CI)
EORTC QLQ-0H15 domains Unadjusted^a^	OH-QoL 8 item scale (q 31-34, 36, 37, 40, 41)	79.8 (78.0-81.5)	85.7 (81.6-89.7)	79.2 (72.8-85.6)	79.7 (76.9-82.6)	77.7 (74.9-80.5)
	Soreness in your mouth (q 35)	19.2 (16.5-21.9)	15.8 (9.1-22.5)	24.4 (14.4-34.4)	14.8 (10.8-18.9)	23.6 (19.0-28.2)
	Sticky saliva (q 38)	38.4 (34.9-41.8)	***21.6 (14.6-28.6****)*	**40.7 (28.1-53.2)**	**43.2 (37.6-48.9**)	**39.1 (33.2-44.9)**
	Sensitive to food and drink (q 39)	24.1 (21.0-27.3)	15.8 (8.7-22.9)	22.0 (11.8-32.1)	25.4 (19.9-31.0)	26.6 (21.5-31.7)
	Have you worn dentures^1^, *n* (%*)	116 (29.0)	21 (36.8)	11 (28.2)	31 (20.1)	53 (35.3)
	- If yes^2^ – problems with an ill-fitting denture	27.6 (21.1-34.0)	**11.7 (1.2-22.1)**	**36.4 (10.9-61.8)**	**31.0 (17.5-44.6)**	**30.0 (20.0-40.0)**
	Information^1^, *n* (%*)	254 (63.5)	30 (52.6)	26 (65.0)	109 (70.8)	89 (59.7)
	- If yes^3^ – satisfied?	70.5 (66.9-74.2)	76.2 (63.6-88.8)	**60.3 (47.6-72.9)**	71.6 (66.3-76.9)	70.5 (64.2-76.8)
EORTC QLQ-0H15 domains Adjusted^b^	OH-QoL 8 item scale (q 31-34, 36, 37, 40, 41)		84.0 (79.3-88.6)	75.8 (70.4-81.3)	80.4 (77.4-83.4)	78.6 (75.8-81.4)
	Soreness in your mouth (q 35)		15.7 (8.3-23.0)	**30.0 (21.4-38.6)**	16.1 (11.3-20.9**)**	20.9 (16.4-25.3)
	Sticky saliva (q 38)		**24.3 (14.6-34.0)**	**43.9 (32.6-55.2)**	**41.1 (34.9-47.4)**	**39.3 (33.5-45.2)**
	Sensitive to food and drink (q 39)		**14.9 (6.2-23.6)**	**25.4 (15.2-35.5)**	**26.6 (20.9-32.3)**	24.8 (19.6-30.1)
	Have you worn dentures^1^, *n* (%)					
	- If yes^2^ – problems with an ill-fitting denture		**10.3 (−6.5-27.0)**	**40.6 (19.7-61.6)**	**29.7 (16.0-43.3**	**30.4 (20.5-40.3)**
	Information^1^, *n* (%)					
	- If yes^3^ – satisfied?		74.8 (63.1-86.5)	**58.6 (46.4-70.9)**	72.5 (66.1-78.9)	70.2 (63.5-76.9)

Scores are linearly transformed to a 0-100 scale. For the OH-QoL scale, higher scores indicate better functioning, while for symptom scales, higher scores indicate more severe symptoms.

^a^ Observed values

^b^ Results are adjusted for age, gender, UICC stage and tumour subsite (oropharynx/oral cavity/others)

**Bolded** numbers indicate clinical relevance i.e. at least one difference of 10 or more points between treatment groups.

*Italic* numbers indicate statistically significant (Tukey post-hoc test < 0.01)

^1^ Four patients with missing information

^2^ Among the patients with “yes”, six patients did not answer “problems with an ill-fitting denture”

^3^ Among the patients with “yes”, three patients did not answer “satisfied?”

* % of answered

*RT* Radiotherapy

*CI* Confidence Interval

*CT* Chemotherapy

*CRT* Chemo-radiotherapy

### Survivor characteristics according to OHRQoL-score

Table 3 shows the characteristics of the survivors according to their OH-QoL scores. The 116 survivors within the poor OHRQoL group were more often females than those with higher OH-QoL scores. They had more advanced disease (UICC stage 3 and 4) at diagnosis and had a worse performance status at the time of the survey (Karnofsky Performance Status ≤70). No difference in the OH-QoL scale score was observed by treatment received.

**Table 3:** Characteristics of the long-term head and neck survivors by score in OH-QoL scale in the EORTC QLQ-OH15

	Lowest* 1/3 OH-QoL score (<75) *n* = 116**	Middle 1/3 OH-QoL score (75-90) *n* = 139**	Highest 1/3 OH-QoL score (>90) *n* = 149**
OH-QoL score median (range)	63 (8-72)	83 (75-88)	96 (92-100)
	*n* (%)	*n* (%)	*n* (%)
Sex^a^			
Male	60 (52)	94 (68)	117 (79)
Female	56 (48)	45 (32)	32 (22)
Age at survey (years)			
Mean [SD] (range)	66 [9] (43-86)	65 [10] (23-89)	67 [10] (23-91)
Country			
Norway	43 (37)	52 (37)	53 (36)
Sweden	20 (17)	31 (22)	50 (34)
Germany	30 (26)	21 (15)	24 (16)
Belgium	14 (12)	29 (21)	20 (13)
Israel	4 (3)	4 (3)	2 (1)
Greece	5 (4)	2 (1)	0 (0)
Living situation			
Together with others	85 (73)	105 (76)	109 (73)
Alone	29 (25)	32 (23)	38 (26)
Missing	2 (2)	2 (1)	2 (1)
Total years of education			
≤10 years	53 (46)	44 (32)	50 (34)
>10 years	61 (53)	95 (68)	98 (66)
Missing	2 (2)	0 (0)	1 (1)
Smoking status			
Never smoker	37 (33)	46 (33)	58 (39)
Former smoker	56 (49)	83 (60)	79 (53)
Current smoker	21(18)	10 (7)	11 (7)
Missing	2 (2)	0 (0)	1 (1)
Tumour subsite			
Oropharynx	48 (41)	53 (38)	69 (46)
Oral cavity	37 (32)	40 (29)	33 (22)
Larynx	7 (6)	13 (9)	20 (13)
Hypopharynx	1 (1)	5 (4)	3 (2)
Nasopharynx	10 (9)	5 (4)	1 (1)
Nasal cavity/sinuses	3 (3)	5 (4)	2 (1)
Parotid/other salivary glands	5 (4)	7 (5)	15 (10)
Unknown primary	5 (4)	11 (8)	6 (4)
Histology			
Squamous cell carcinoma	101 (87)	122 (88)	130 (87)
Other	13 (11)	14 (10)	19 (13)
Missing	2 (2)	3 (2)	0 (0)
UICC stage^b^			
I	12 (10)	20 (14)	44 (30)
II	17 (15)	22 (16)	23 (15)
III	31 (27)	38 (27)	44 (30)
IV	47 (41)	55 (40)	37 (25)
Missing	7 (6)	3 (2)	1 (1)
Treatment			
Surgery	11 (10)	14 (10)	32 (22)
RT	13 (11)	13 (9)	15 (10)
CRT	40 (34)	59 (42)	56 (38)
Surgery+RT±CT	52 (45)	53 (38)	46 (31)
Karnofsky Performance Status			
100	31 (27)	64 (46)	98 (66)
80 and 90	65 (56)	66 (48)	45 (30)
≤70	18 (16)	8 (6)	5 (3)
Unknown	2 (2)	1 (1)	1 (1)
Charlson comorbidity index			
0	67 (58)	83 (60)	96 (64)
1	27 (23)	31 (22)	27 (18)
2	11 (9)	13 (9)	17 (11)
≥3	11 (9)	12 (9)	9 (6)
Current evidence of disease			
Yes	3 (3)	0 (0)	1 (1)
No	112 (97)	138 (100)	147 (99)
Missing	1 (1)	1 (1)	1 (1)
Time since diagnosis (years)			
5 to > 8 years	41 (35)	57 (41)	44 (30)
8 to > 10 years	38 (33)	43 (31)	66 (44)
≥ 10 years	37 (32)	39 (28)	39 (26)

^a^ Chi-square test for independence *p* < 0.005

^b^ Jonckheere-Terpstra test for trend *p* < 0.005

* lowest score in OH-QoL scale in the EORTC QLQ-OH15 means poorest OHRQoL

**The tertiles aimed to be equally sized, but the groups were not perfectly equal due to ties

*RT* Radiotherapy

*CT* Chemotherapy

*CRT* Chemo-radiotherapy

*SD* Standard deviation

*UICC* Union for International Cancer Control

The logistic regression analyses also showed that female survivors had a higher likelihood of low OHRQoL compared to men (OR 2.6, 95% CI 1.6-4.0) (Table 4). This finding remained clinically significant after multivariable adjustment (aOR 2.6, 95% CI 1.34-5.22). No other patient/tumour characteristics were associated with OHRQoL.

**Table 4:** Logistic regression analyses of factors associated with poor OHRQoL in long term head and neck cancer survivors, *n* = 404^ab^

	Univariable analyses			Multivariable analyses		
	OR	95% CI	*p*-value	aOR^c^	95% CI	*p*-value
Age at survey (per 10 years)	0.99	0.80-1.24	0.99	1.01	0.79-1.29	0.92
Sex (female vs. male)	**2.56**	**1.63-4.00**	**<0.001**	**2.64**	**1.34-5.22**	**<0.01**
Tumor subsite						
Oropharynx	Ref.					
Oral cavity	1.29	0.77-2.16	0.34	1.17	0.70-1.96	0.54
Others	0.85	0.50-1.43	0.54	0.77	0.45-1.31	0.33
Treatment						
Surgery	Ref.					
RT	1.94	0.77-4.92	0.16	2.54	0.92-7.00	0.07
CRT	1.45	0.69-3.08	0.33	1.89	1.03-3.47	0.04
Surgery+RT±CT	2.20	1.05-4.60	0.04	2.36	0.62-9.05	0.21
UICC stage						
I & II	Ref.					
III	1.34	0.76-2.38	0.32			
IV	1.81	1.07-3.08	0.03			
Time since diagnosis						
5 to >8 years	Ref.					
8 to >10 years	0.86	0.51-1.44	0.56	0.85	0.53-1.35	0.48
>= 10 years	1.17	0.69-1.99	0.57	1.21	0.79-1.86	0.39

Abbreviations: *OR* Odds ratio; *aOR* Adjusted Odds Ratio; *CI* Confidence interval; *RT* Radiotherapy; *CRT* Chemoradiotherapy

Bold: p <0.01

^a^ Survivors were categorized by tertiles of the OH-QoL scale score in EORTC QLQ-OH15 (low: OHRQoL score <75, middle and high combined: OHRQoL score ≥75).

^b^ Unknown UICC stage (*n* = 11) was omitted from analysis

^c^ Adjusted for age, sex, tumor subsite, treatment and time since diagnosis, and clustered by country

### Toxicity

The most common clinician rated oral toxicity (grade 1-3) was dry mouth (77%), while they reported that 59% had dysphagia, 23% had trismus, 19% had oral pain and 7% had osteonecrosis of the jaw (Table 5). The survivors with poor OHRQoL had more often problems with dysphagia, trismus, osteonecrosis of the jaw, oral pain and dry mouth than survivors with better OHRQoL. These associations were clinically significant in all the selected toxicities (dysphagia, trismus, oral pain and dry mouth) except for osteonecrosis of the jaw.

**Table 5:** Toxicity of the long-term head and neck survivors by score in OH-QoL scale in the EORTC QLQ-OH15

CTCAE	Total *n *= 404	Lowest* 1/3 OH-QoL score (<75) *n *= 116**	Middle 1/3 OH-QoL score (75-90) *n *= 139**	Highest 1/3 OH-QoL score (>90) *n *= 149**
Dysphagia^a^				
Not present (0)	160 (40)	20 (17)	48 (35)	92 (62)
Mild (1)	130 (32)	33 (28)	56 (40)	41 (28)
Moderate (2)	95 (24)	51 (44)	31 (22)	13 (9)
Severe (3)	12 (3)	10 (9)	2 (1)	0 (0)
Missing	7 (2)	2 (2)	2 (1)	3 (2)
Trismus^a^				
Not present (0)	301 (75)	71 (61)	105 (76)	125 (84)
Mild (1)	66 (16)	21 (18)	25 (18)	20 (13)
Moderate (2)	30 (7)	20 (17)	8 (6)	2 (1)
Severe (3)	1 (0)	1 (1)	0 (0)	0 (0)
Missing	6 (1)	3 (3)	1 (1)	2 (1)
Osteonecrosis of the jaw				
Not present (0)	368 (91)	97 (84)	131 (94)	140 (94)
Mild (1)	7 (2)	4 (3)	1 (1)	2 (1)
Moderate (2)	13 (3)	6 (5)	3 (2)	4 (3)
Severe (3)	10 (2)	6 (5)	3 (2)	1 (1)
Missing	6 (1)	3 (3)	1 (1)	2 (1)
Oral pain^a^				
Not present (0)	319 (79)	75 (65)	108 (78)	136 (91)
Mild (1)	62 (15)	24 (21)	29 (21)	9 (6)
Moderate (2)	17 (4)	14 (12)	1 (1)	2 (1)
Severe (3)	1 (0)	1 (1)	0 (0)	0 (0)
Missing	5 (1)	2 (2)	1 (1)	2 (1)
Dry mouth^a^				
Not present (0)	93 (23)	4 (3)	24 (17)	65 (44)
Mild (1)	184 (46)	42 (36)	70 (50)	72 (48)
Moderate (2)	116 (29)	62 (53)	43 (31)	11 (7)
Severe (3)	7 (2)	6 (5)	1 (1)	0 (0)
Missing	4 (1)	2 (2)	1 (1)	1 (1)

^a^ Jonckheere–Terpstra test for trend p < 0.005

* lowest score in OH-QoL scale in the EORTC QLQ-OH15 means poorest OHRQoL

**The tertiles aimed to be equally sized, but the groups were not perfectly equal due to ties

*CTCAE* Common Terminology Criteria for Adverse Events

### HRQoL results by EORTC QLQ-C30

HRQoL, as measured with EORTC QLQ-C30 questionnaire, are presented by treatment groups in Supplementary Table 1. Survivors in the surgery alone group had significantly less fatigue than survivors who had received surgery in combination with (chemo) RT.

## Discussion

Good oral health is characterized by the absence of pain, discomfort and disease in the oral cavity [35]. As oral health is an integral part of general health and well-being, disturbance of vital functions in the head neck region and experience of oral late effects result in impairment of OHRQoL [36]. To our knowledge, this is the first study to describe long-term OHRQoL in an international cohort of HNC survivors with a median follow-up time of more than eight years post-treatment. The findings of this study highlight the importance and need for improved follow-up of the oral health of HNC survivors many years after initial treatment. The recently published survivorship care recommendations of the European Head and Neck Society emphasize the importance of long-term follow up care [37]. Knowledge of OHRQoL may help both clinicians and health authorities to prioritize follow-up strategies between groups of patients and evaluate the effect of interventions [38]. It also emphasizes the crucial role dental health personnel play in the care of HNC survivors, and the importance of implementing long-term oral health management into survivorship care planning. Regular dental visits and specialized oral health care should be an integral part of survivorship programs. Furthermore, health professionals need knowledge on how to recognize, prevent and manage oral late effects. There is a need for a multidisciplinary approach, involving oncologists, HN surgeons and dental professionals to recognize, prevent and manage oral late effects in HNC survivors. Implementing OHRQoL assessment as routine in the treatment of HNC patients could better inform the multidisciplinary teams and lead to more personalized treatment plans, improved patient-provider communication, earlier identification of supportive care needs and reduction of long-term burden of treatment toxicities [9]. This knowledge is essential for providing comprehensive care and improve HNC survivors' OHRQoL long-term.

Our study confirmed that oral late effects persist in many long-term HNC survivors and affect OHRQoL negatively. Many oral late effects have been shown to persist for years after treatment [23, 40]. In the EORTC 1629 project, dry mouth was reported as the most frequent serious long-term problem by 46% of the HNC survivors [40]. In a study by Hammerlid and coworkers many side effects of HNC treatment resolved after three years, but problems related to oral health, including dry mouth, teeth problems and trismus persisted [22]. Among a Swedish/Norwegian population of long-term pharyngeal cancer survivors, dry mouth was reported as the most burdensome symptom after five years follow-up [39].

As expected, most participants in the current study were males, which is in accordance with the HNC epidemiology. However, our findings revealed gender differences in long-term OHRQoL with female survivors being more likely to report poor OHRQoL. This difference persisted after adjusting for other factors and is in line with other studies suggesting that women may have a higher risk of developing late effects [41]. Huynh et al found that long-term dysphagia also was significantly associated with being female in a population of 239 long-term HNC survivors [42]. It has also been shown that in reference populations females reported more symptoms and more impaired HRQoL than males [43]. However, a meta-analysis of oral cancer patients done by Yuwanati and coworkers found that males had poorer OHRQoL than females [44]. Exact reasons for this difference could not be ascertained, but a tendency to more frequent dental visits by females could be one of them. Some studies did also have less/fewer female inclusions in their study populations due to HNC epidemiology. The general mechanisms behind gender differences are not fully understood [45], and the contributing factors are complex and could be attributed to genetic, biological and behavioral differences [46, 47]. This observation warrants further investigation and assessment of OHRQoL long-term.

As expected, our study found that HNC survivors treated for advanced disease were significantly more likely to report poor OHRQoL than those treated for local disease. This is in line with previous data [17] and may be explained by the fact that patients with locally advanced disease receive more intense treatment and have a higher risk of developing disease and treatment related sequela. Our study revealed no other patient or tumour characteristics associated with poor OHRQoL. Based on previous literature we would have expected poorer OHRQoL outcomes in the oral cavity– and oropharynx groups compared to larynx cancer [16], but this might be attributed to the relatively small group of larynx cancer patients (10%) included in the study. Some studies have shown that older patients report poorer OHRQoL compared to younger patients [44].

It was unexpected that we found no clinically significant differences in OHRQoL between the treatment groups, as previous research has reported such notable differences [18, 28, 48]. One possible explanation might be that our study is the first to examine this relationship more than five years post treatment, suggesting that passage of time may influence the outcome. Nevertheless, this need to be confirmed by further research. The EORTC 1629 study found that multimodal treatment was associated with worse long-term HRQoL compared to single treatment modalities [28]. Another study found that oral cancer survivors who received post-operative chemo-radiation had worse OHRQoL after six months compared to surgery alone [18]. There is no information whether this difference persisted in the long-term. The previous mentioned meta-analysis of OHRQoL in oral cancer patients showed that the patients had poorer OHRQoL compared to healthy controls, irrespective of type of treatment. However, patients treated with RT had poorer OHRQoL outcomes than those who underwent surgery alone, supporting that additional treatment of either RT or chemotherapy (or a combination) worsen the OHRQoL [46].

Although we did not find a difference in OHRQoL between treatment groups, survivors in the surgery alone group reported less sticky saliva, reduced sensitivity to food and drink and fewer problems with ill-fitting dentures. Similar findings have previously been reported in a Swedish/Norwegian population of oral cancer survivors five years after treatment [12].

The survivors who reported poor OHRQoL had a high prevalence of dysphagia, trismus, osteonecrosis of the jaw, oral pain and dry mouth. This is in line with a newly published study [48]. A systematic review on long-term toxicity among HNC patients more than five years post-treatment found some evidence that survivors had long-term problems with dry mouth, dysphagia and hearing [49].

This study is strengthened by the large sample size and use of the well-established, validated EORTC QLQ-OH15 questionnaire to ascertain OHRQoL from a patient perspective. By incorporating perspectives from survivors across multiple countries, this study provides an international, comprehensive and relevant understanding of long-term OHRQoL in HNC survivors. There are several validated questionnaires to evaluate OHRQoL in this patient group. Previous studies have used EORTC QLQ-H&N35 which was designed to measure HRQoL among HNC patients [50]. This may be due to lack of a specific tool to evaluate the OHRQOL in cancer patients until the more recently developed EORTC QLQ-OH15 [8]. EORTC QLQ-OH-15 was developed to measure OHRQoL primarily in general cancer groups but is also relevant to HNC patients and include important aspects on oral health and topics such as pain, sensitivity to food and drink, information received and use of dentures. Many studies have also evaluated OHRQoL with the Oral Impact on Daily Performances (OIDP) questionnaire and the Oral Health Impact Profile-14 (OHIP-14) [51, 52]. Deana and coworkers provided a systematic review evaluating OHRQoL instruments available for older adults and their findings supported using EORTC QLQ-OH15 as a specific instrument to assess OHRQoL in cancer patients [53].

The cross-sectional study design and lack of baseline measurements prevent analyses of changes in OHRQoL over time, and we have no data about the survivor`s dental care during follow-up. Prospective longitudinal studies would be preferable but are resource demanding. A few studies have done/carried out before treatment/baseline measurements of OHRQoL with two years follow-up after treatment [21, 54, 55]. As far as we know, no longitudinal studies with more than five years follow-up for this patient group has been published. Our study has no control group, but some studies have compared OHRQoL of HNC survivors to general population cohorts, showing that HNC treatment correlate with poor OHRQoL [46, 56]. Our study population may be subject to selection bias, which we are unable to fully account for. We were unable at some sites to collect information about survivors who declined to participate. It might be that survivors with the most problems were more likely to participate in the study than those without issues, as the examination might seem more relevant to them, but the opposite can also be true as survivors with most problems might have found it too burdensome to participate in the study because it required them to travel to the clinic.

## Conclusion

This study provides a comprehensive overview of long-term OHRQoL in HNC survivors. Poor long-term OHRQoL may be associated with high grade of toxicity, female sex and advanced disease suggesting that these groups of survivors may require additional support and tailored interventions to improve their long-term OHRQoL. The survivors with poor OHRQoL had more often problems with dry mouth, dysphagia, trismus, oral pain and osteonecrosis of the jaw. Including PROMs in routine clinical care is valuable for identifying patients` late effects and oral complaints, thereby enabling tailored symptom management and follow-up care. The findings highlight the significant and enduring impact of HNC and its treatment, and provides a basis to suggest that multidisciplinary, tailored and long-term oral supportive care strategies are essential to optimize outcomes for the growing population of HNC survivors.

## Data Availability

No datasets were generated or analysed during the current study.

## Declarations

**Ethical considerations**

All participants gave written informed consent before enrolment in the study, which was conducted in accordance with the principles of the Declaration of Helsinki. The ethical approval at the coordinating center in Mainz, Germany, was granted by the Landesärztekammer (Medical Association) Rhineland-Palatinate (No. 2018-13579) and was obtained at each site in accordance with local regulations.
