# Correction to: Oral health‑related quality of life among long‑term head and neck cancer survivors: a multinational study

**DOI:** 10.1007/s00520-025-09991-9

**Published:** 2025-10-01

**Authors:** Kristine Løken Westgaard, Cecilie Delphin Amdal, Katherine J. Taylor, Ragnhild Sørum Falk, Kristin Bjordal, Susanne Singer, Eva Hammerlid, Max Krüger, Carmen Stromberger, Orlando Guntinas‑Lichius, Fréderic Duprez, Noa Stempler, Noam Yarom, Erofili Papadopoulou, Bente Brokstad Herlofson

**Affiliations:** 1 Department of Oral Surgery and Oral Medicine, Faculty of Dentistry, University of Oslo, Postbox 1109 Blindern, N‑0317 Oslo, Norway; 2 Department of Otorhinolaryngology, Division for Head, Neck, and Reconstructive Surgery, Unit of Oral and Maxillofacial Surgery, Oslo University Hospital, Oslo, Norway; 3 Department of Oncology, Oslo University Hospital, Oslo, Norway; 4 Research Support Services, Oslo University Hospital Oslo, Oslo, Norway; 5 Institute of Medical Biostatistics, Epidemiology, and Informatics, University Medical Centre Mainz, Langenbeck, Germany; 6 Faculty of Medicine, University of Oslo, Oslo, Norway; 7 Department of Quality of Life in Oncology, Comprehensive Cancer Center Mecklenburg‑Vorpommern (CCC‑MV), University Medical Centre Rostock, Campus Rostock, Rostock, Germany; 8 Department of Otorhinolaryngology‑Head and Neck Surgery, Institute of Clinical Sciences, Sahlgrenska Academy at University of Gothenburg, Sahlgrenska University Hospital, Gothenburg, Sweden; 9 Department of Oral and Maxillofacial Surgery ‑ plastic Surgery, University Medical Centre Mainz, Mainz, Germany; 10 Department of Radiation Oncology, Charité-Universitätsmedizin Berlin, Corporate member of Freie Universität Berlin, Humboldt-Universität zu Berlin Germany, Berlin, Germany; 11 Berlin Institute of Health, Germany, Berlin, Germany; 12 Department of Otorhinolaryngology, Jena University Hospital, Jena, Germany; 13 Department of Radiotherapy‑Oncology, Ghent University Hospital, Ghent, Belgium; 14 Faculty of Medicine and Health Sciences – Department Human Structure and Repair, Ghent University, Ghent, Belgium; 15 Oral Medicine Unit, Sheba Medical Center, Tel Hashomer, Israel; 16 The Goldschleger School of Dental Medicine, Faculty of Medical and Health Sciences, Tel Aviv University, Tel Aviv, Israel; 17 Department of Oral Medicine and Pathology and Hospital Dentistry, Dental Oncology Unit, Dental School, National and Kapodistrian University of Athens, Athens, Greece

**Correction to: Supportive Care in Cancer (2025)**

10.1007/s00520-025-09930-8

The published article “Oral health‑related quality of life among long‑term head and neck cancer survivors: a multinational study”.

Regrettably, the initial version of this paper contains some inaccuracies. Reference 4 is misidentified. The accurate reference 4 is provided below, and as a result, the subsequent citations and references will need to be renumbered.

4. https://qol.eortc.org/quality-of-life/

The Publisher regrets this error.

The original article has been corrected.

